# Porto-biliary fistula as an uncommon cause of haemobilia: A case report and literature review

**DOI:** 10.1016/j.ijscr.2022.107635

**Published:** 2022-09-09

**Authors:** Valentina Galvis, Daniela Ayala, Juliana González T, Carlos Eduardo Rey Chaves, Danny Conde, German Gomez, Juan Carlos Sabogal Olarte

**Affiliations:** a School of Medicine, Universidad del Rosario, Bogotá D.C. 111711, Colombia; b Department of Hepatobiliary and Pancreatic Surgery, Hospital Universitario Méderi, Bogotá 111711, Colombia; c School of Medicine, Pontifical Xavierian University, Bogotá, Colombia; d Clinica del Country, Bogota, Colombia

**Keywords:** Haemobilia, Porto-biliary fistula, Bile duct, Laparotomy

## Abstract

**Background:**

Haemobilia is a rare cause of gastrointestinal bleeding. It can be related to iatrogenic injuries, inflammatory diseases, and, more recently, postoperative, or post-procedure complications. Porto-biliary fistula is an uncommon case of haemobilia and has been related to iatrogenic injury or chronic inflammatory processes. To date, less than 30 cases of Porto-biliary fistula have been reported.

**Case presentation:**

We present a 53 years-old woman with a history of biliary obstruction due to a choledochal cyst that required hepaticojejunostomy with evidence of anastomotic stricture. A percutaneous transhepatic biliary drainage (PTBD) was performed, with 3 failed attempts of percutaneous dilatation. A new hepaticojejunostomy was completed, however, 45 days later the patient presented to the emergency room with haemobilia and secondary hemodynamic instability. An emergency damage control laparotomy was performed, achieving bleeding control. In the second procedure, there is evidence of an ulcerative injury of the biliary tract secondary to a Porto-biliary fistula.

**Conclusion:**

Porto-biliary fistula is an entity that cannot be ruled out in cases of haemobilia, especially in cases with a history of bile duct surgical or percutaneous procedures. The prognosis is usually good if multidisciplinary management is performed, and the source of the bleeding is identified early.

## Highlights

• Portobiliary is a rare condition with increased risk of mortality.
• Clinical suspicion should be present in patients with multiple procedures in the bile tract.
• Multidisciplinary management increases the rate of successful management.
• Ureteroscopy could be an alternative to choledocoscopy in emergency context.

## 1 Introduction

Haemobilia was described for the first time in 1654 by Francis Gilsson [1]. It refers to the extravasation of blood through the bile duct [2]. Haemobilia is evidenced in liver trauma (55 %), inflammatory diseases (28 %), vascular malformations (11 %), bile duct tumors, and, in less proportion, periampullary tumors (6 %) [3], [4]. However, its rate has increased with minimally invasive procedures performance and pancreatic localized procedures [5]. Clinical presentation is very unspecific and depends on the clinical condition of the patient, representing a diagnostic challenge. The symptoms related to bleeding such as hematemesis, melaena, hematochezia, or hemodynamic instability have been described [1], [6].

Porto-biliary fistula is an uncommon cause of haemobilia. It is a complication after bile duct procedures and manipulation [7]. It is a rare entity and the literature on this topic is restricted to case reports [7], [8], [9]. To date, less than 30 cases have been described. Due to its low incidence and unspecific clinical presentation, Porto-biliary fistula represents a diagnostic and therapeutic challenge that can be life-threatening [8], [9].

## 2 Case presentation

After ethical and institutional approval, with informed consent filled, and following SCARE guidelines [16]. We present a 53-years-old female with a medical history of biliary obstruction due to a choledochal cyst, requiring hepaticojejunostomy. 20 years after the surgery, the patient presented anastomotic stenosis, which required a percutaneous transhepatic biliary drainage (PTBD) with multiple failed attempts of percutaneous dilatation, wherefore it was indicated again a hepaticojejunostomy, with apparently no incidences.

On the 45th postoperative day, the patient presented to the emergency room due to severe abdominal pain associated with bleeding through the PTBD. A percutaneous cholangiography through the PTBD catheter was performed with evidence of a connection between the biliary tract and the portal system. Therefore, she was referred to our institution.

**Fig. 1:**
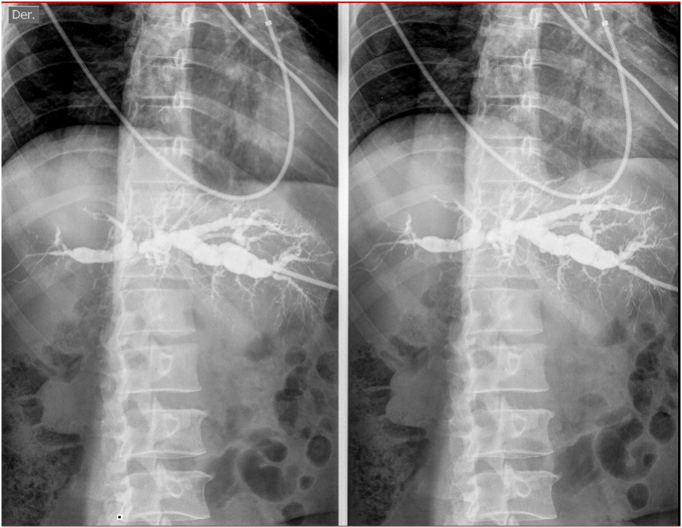
Cholangiography.

At the initial evaluation, the patient was stable, however, the blood test evidenced severe anemia that requires red blood cell transfusion. An institutional percutaneous cholangiography through the PTBD catheter (Fig. 1) and a magnetic resonance portography were performed with no evidence of fistulous tracts. Due to the diagnostic challenge, the initial management was the withdrawal of the percutaneous transhepatic biliary drainage. After one day of favorable evolution with no signs of upper gastrointestinal bleeding, the patient was discharged.

Twenty-four hours after being discharged, the patient was taken to the emergency room due to massive hematemesis. To physical examination the patient was unstable. Blood tests evidenced a 3 g/dL decrease in red blood cells in less than 24 h. An urgent esophagogastroduodenoscopy (EDGE) was performed, nevertheless, the source of the bleeding could not be identified.

The patient had a torpid evolution with substantial hemodynamic instability that required vasopressor support. Blood tests evidence persistent bleeding with a deterioration of blood parameters, decreasing hemoglobin levels as low as 4 g/dL. With suspected Porto-biliary fistula, the patient was taken to surgery.

An urgent laparotomy by midline incision - damage control surgery - was performed, and vascular control was performed using a Pringle maneuver. The previous hepaticojejunostomy was taken down, and an exploration of the bile duct was performed, identifying bleeding from the proximal bile ducts. An 18-French Foley catheter was inserted 2.5 cm into the proximal bile duct and was used to stop the bleeding (Inflated with 5 mL of ringer lactate), achieving hemodynamic stability. Bile ducts were canalized and exteriorized using a nelaton catheter for left and right hepatic ducts.

**Fig. 2:**
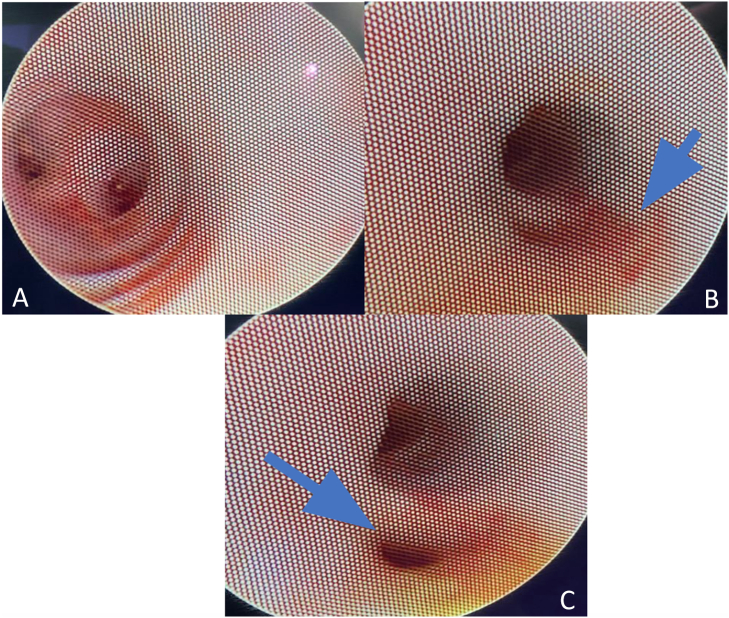
A. Endoscopic view of left and right hepatic ducts. B. Endoscopic visualization of wall erosion at the intrahepatic bile ducts. C Endoscopic visualization of the portobiliary fistula.

After 24 h, an emergency re-exploration was required due to persistent bleeding through the nelaton catheters. During this time using a flexible ureteroscope, an ulcerative injury was identified. It was identified on the medial side of the left hepatic duct, straight after the common hepatic duct. The injury had traces of fibrin and erythema with active bleeding. Dissection of the common hepatic duct was performed, finding a communicating vessel between the biliary tract and the portal system (Fig. 2). It was ligated, and Blake's drainage was left. Intensive care unit was required. On the 7th day, the patient was discharged, and with favorable evolution, Blake's drainage was left in order to evaluate signs of bleeding. After 3 months, the bile duct was reconstructed by a new hepaticojejunostomy without incidences. No postoperative complications have been seen on 90 days of follow-up.

## 3 Discussion

Haemobilia remains an uncommon cause of upper gastrointestinal bleeding, with unknown incidence and prevalence [7], [8]. To date, less than 30 cases have been described in the worldwide literature. The most common causes related to haemobilia are iatrogenic injuries (65 %) after percutaneous liver biopsy, following laparoscopic cholecystectomy, associated with percutaneous biliary drainage placement, following ERCP, or after transjugular portosystemic shunt placement (TIPS), followed by accidental trauma (15 %) and inflammatory diseases (9 %) [6], [10]. These procedures could generate an increased inflammatory response near to the vessels (with the formation of small pseudoaneurysms in some cases) and due to bile fluid contact an endothelium injury [1], [4], [11]. Rare cases have been related to chronic obstruction of the bile ducts [12].

According to Zhou et al., haemobilia represents 3 % of all complications after liver biopsy [13]. Also, Rivera et al. evidenced a 0.7 to 2.2 % increased risk of haemobilia after PBTD in patients with biliary obstruction [14]. After undergoing hepatic-bilio-pancreatic procedures, haemobilia can be produced by abnormal communication between a vessel and the bile tract [6], [10]. It can be an arterial cause, due to a communication of the biliary tract with the hepatic artery, a branch of the common hepatic artery, or a venous cause, with direct communication with the portal vein, which drains this system [11], [12].

Clinical presentation is unspecific. The Quincke's triad has been reported in 22–35 % of the cases [2], [6]. It was described for the first time in 1870 by Heinrich Quincke and includes right upper quadrant abdominal pain, jaundice, and gastrointestinal bleeding that is persistent in time [2], [12], [16]. Other nonspecific symptoms could be associated.

This entity can be life-threatening due to massive bleeding that can lead to a hypovolemic shock. Initial blood tests can show nonspecific findings like anemia, elevated liver enzymes, and elevated acute-phase reactants [1], [2]. As the diagnosis remains a challenge, clinical suspicion is important to manage this pathology before developing complications [2], [13], [14], [15]. The diagnosis requires a multidisciplinary and integrative approach, notwithstanding it is based on clinical features, it may require a combination of endoscopic and imaging modalities [16]. CT has become a common option for the evaluation of gastrointestinal bleeding and CT angiography has demonstrated a sensitivity and specificity of up to 90–95 %, if the bleeding rate is between 0.3 and 0.5 mL/min, to identify the source [17], [18]. Esophagogastroduodenoscopy (EDGE) can show a direct visualization of active bleeding through the bile ducts, confirming the diagnosis [16].

Despite advances in imaging, angiography remains the gold standard diagnostic method, due to its diagnostic and therapeutic ability in stable patients [18]. The goal of treatment is to stop bleeding and ensure bile flow. In selected cases, percutaneous endovascular management could be performed [19], [20]. Some authors such as Chaitowitzp et al., Peynircioglu et al. have reported the use of stent grafts to obliterate and resolve the fistula in stable patients with positive outcomes [21], [22].

In the present case, haemobilia resulted from a Porto-biliary fistula, with massive bleeding, leading to hemodynamic instability. Due to the patient's clinical condition, the diagnosis was made intraoperatively by direct visualization through ureteroscopy through a damage control surgery. Subsequently, once the patient reached hemodynamic stability, with better control of bleeding, reconstruction of the biliary tract was performed with a new hepaticojejunostomy, no complications were reported to date.

## 4 Conclusion

Porto-biliary fistula remains to be an uncommon cause of haemobilia. Special attention in patients with chronic bile obstruction, malignancy, or persistent inflammatory biliary diseases is important to early diagnose and treat the condition. Multidisciplinary management should be performed to achieve the diagnostic challenge. The high clinical suspicion and prompt use of diagnosis modality are paramount in reducing morbidity and mortality.

## Consent

All the including participants of the study, fill the appropriate informed consent. Upon request we will provide the form.

## Availability of data and materials

The datasets used and/or analyzed during the current study are available from the corresponding author on reasonable request.

## Ethical approval

Ethical approval of institutional committee was obtained.

## Funding

Authors do not declare any financial conflict.

## Guarantor

Carlos Rey.

## Research registration number

Do not apply.

## CRediT authorship contribution statement

DA: Manuscript writing, critical revision of the manuscript, data analysis.
VG: Data analysis, manuscript writing.
JG: Data analysis, manuscript writing.
CR: Manuscript writing, critical revision of the manuscript, data analysis.
DC: Manuscript writing, critical revision of the manuscript, data analysis.
JS: Manuscript writing, critical revision of the manuscript, data analysis.

## Declaration of competing interest

Authors do not declare any conflict of interest.
